# Views of English Pharmacists on Providing Public Health Services

**DOI:** 10.3390/pharmacy3040154

**Published:** 2015-10-13

**Authors:** Catherine Dewsbury, Ruth M Rodgers, Janet Krska

**Affiliations:** Medway School of Pharmacy, The Universities of Greenwich and Kent, Central Avenue, Chatham Maritime, Kent ME4 4TB, UK; E-Mails: c.dewsbury@kent.ac.uk (C.D.); r.m.rodgers@kent.ac.uk (R.M.R.)

**Keywords:** community pharmacy, public health, commissioning, primary care, pharmacist opinions

## Abstract

Locally-commissioned pharmacy public health services have developed in England over the last 20 years. Few studies have sought pharmacist views on commissioning and provision of public health services in general. This study sought views of community pharmacists (*n* = 778) in 16 areas of England on services provided, decisions about services, support, promotion and future developments, using a postal questionnaire with two reminders. Response rate was 26.5% (206). Funded public health services provided most frequently were: emergency contraception (71%), smoking cessation (62%), and supervised drug consumption (58%). Blood pressure monitoring was provided by 61% and was considered to be one of the services pharmacists perceived as being most valued by customers, but was not National Health Services (NHS)-funded. Motivation for providing public health services was professional not financial, particularly from those working in independent pharmacies. Only 35% were personally involved in deciding which services to deliver, and fewer than 20% based decisions on local public health reports. Pharmacists had positive attitudes towards providing public health services, but mixed views on support for services and their promotion. Most thought services would increase in future, but were concerned about commissioning. Both national and local support is needed to ensure future commissioning of pharmacy public health services.

## 1. Introduction

A succession of governments in England has acknowledged the potential contribution of community pharmacy in supporting public health [1,2,3,4,5]. Public health services are also supported by pharmacy contractor representatives and professional bodies [6,7,8,9,10]. A wide range of pharmacy public health services have developed over the last 20 years, with public health being embedded into the revision of the National Health Services (NHS) pharmacy contractual framework in 2005 [11]. Since then, enhanced services, such as needle exchange schemes and supervised consumption of medicines for drug misusers, minor ailment schemes, smoking cessation services and some sexual health services have become widespread nationally, but were commissioned by local health organisations—Primary Care Trusts (PCTs).

Pharmacists working within PCTs contributed to the decisions of these commissioning organisations to develop specific services, based on local needs. The need for services was identified by PCT staff using information provided in Local Health Profiles, published by Public Health Observatories, reports from Directors of Public Health (DPH) and a Joint Strategic Needs Assessment (JSNA), which incorporates information from a Pharmaceutical Needs Assessment (PNA). Each area would thus be expected to commission different services, dependent on the health needs of the local population.

The commissioners normally negotiate the terms of service provision with a Local Pharmaceutical Committee (LPC), which represents the community pharmacy contractors in the area, however, since the 2005 revision of the contractual framework, there is no longer a requirement to do so. A study showed that despite this, both Directors of Public Health and pharmacists working in PCTs thought the revised contractual framework would strengthen pharmacists’ public health role, but felt this could be constrained by lack of funding [12]. The implementation of the revised contract was evaluated two years after its introduction, and showed that over half of community pharmacies had increased their public health activities: most were offering health promotion and over 85% were providing at least one public health service, most frequently smoking cessation (44%), medicine supply through patient group directions (written instructions for the supply or administration of medicines to groups of patients who may not be individually identified before presentation for treatment) (42%) and supervised administration of medicines (39%) [11]. Other work suggested that services were provided with differing frequencies depending on location and ownership [13].

Since then, the Healthy Living Pharmacy (HLP) concept was developed, which provides a framework for commissioning public health services through three levels of increasing complexity and the expertise required of pharmacy staff [14]. Early work evaluating this programme suggested that HLPs saw more clients for public health services than other pharmacies in the same geographical area [15] and a larger evaluation, published in 2013, found that HLPs delivered a wider range of services than other pharmacies [16].

However, major changes to the structure of primary care in England in April 2013 resulted in the abolition of PCTs, and responsibility for local public health was moved to local government. Commissioning of primary care services thus became the responsibility of the NHS Commissioning Board (known as NHS England) and locally-based Clinical Commissioning Groups, the latter comprising mostly general practitioners [17]. This re-structuring was anticipated to result in changes to the commissioning and provision of pharmacy public health services, which could either suffer or be enhanced. This study determined the views of community pharmacists on public health services in general and their perceptions of the future, immediately prior to the implementation of this significant change.

Much research has shown that community pharmacists have positive attitudes towards providing specific public health services, but studies suggest that while most pharmacists see these as important and part of their role, they are considered secondary to medicine-related roles [18]. Moreover, most studies seeking pharmacist views look at individual public health services [18,19] with very little work having obtained views on public health services in general and/or policies driving these [13]. The changes in commissioning mechanisms presented an opportunity to seek the views of English pharmacists on provision of public health services in general, to form a baseline for future comparison and to enable comparison with previous work [13].

It may be anticipated that pharmacists working in areas where many different services are commissioned could have more positive views of their role in public health and that pharmacists with managerial responsibilities or pharmacy owners may welcome the opportunity to increase income through these commissioned services. The aims were thus: (i) to obtain information about public health service provision and the views of community pharmacists in England on providing these services; and (ii) to assess whether service provision and views differed depending on the pharmacy ownership, pharmacist role and the number of PCT-reported commissioned pharmacy public health services.

## 2. Methods

The study was approved by a University Research ethics committee and conducted in November 2012, prior to implementation of the NHS reorganisation in April 2013.

### 2.1. Questionnaire Development

A random sample of 20 pharmacists from one PCT, which was randomly selected from those reported to be providing two or three public health services, were invited to take part in a telephone interview. Eleven agreed and were interviewed, the findings of which informed the content of the questionnaire. The questionnaire was piloted by postal distribution to the remaining pharmacists in this area, who were also asked to evaluate the content, ease of use and time taken to complete.

The final questionnaire covered service provision, selection of services and views on public health service commissioning, provision and importance. Questions on service provision included: a list of 26 potential services mentioned in interviews from which respondents could select those they considered as public health services, those they provided and those which were NHS-funded; reasons for public health service delivery in general and service selection, using options derived from interview data; open-ended questions seeking the three public health services pharmacists viewed as most valued by customers and any other services respondents wished to provide. Selection of services was determined using: closed questions seeking reasons for providing public health services; perceptions of who made decisions about service provision; awareness and use of four local (PNA, JSNA, DPH report and Local Health Profile) and two national documents (Pharmacy in England 2008, Choosing Health through Pharmacy) in selecting services. Views on public perceptions, promotion, support and the future of pharmacy public health services, were sought using 5-point semantic differential scales, plus four statements accompanied by 5-point Likert response options, covering the areas raised by pharmacist in interviews. Demographic data collected covered pharmacist gender, years qualified, role in pharmacy, plus pharmacy ownership, location and HLP status.

### 2.2. Questionnaire Distribution

PCTs were selected from annual returns of NHS General Pharmaceutical Services provision for the year April 2010–March 2011 [20], excluding those not providing an annual return. The remainder were stratified dependent on whether 1, 2, 3 or ≥ 4 of the following services were reported as being commissioned: minor ailment schemes; needle exchange services; services under Patient Group Direction (PGD); screening; stop smoking; supervised administration of medicines. NHS data were not available for any other pharmacy public health services. A 10% sample from each stratum was randomly selected using a random number generator. All pharmacies in each PCT were sent a questionnaire by post, accompanied by an information sheet, plus a freepost envelope for return of the completed questionnaire. One postal reminder was sent and a reminder telephone call made to non-responders.

### 2.3. Data Analysis

Data were analysed using SPSS version 20. Pharmacist roles were collapsed into owner/superintendent and manager/second pharmacist/locum to enable data analysis, which used Chi-squared tests and t-tests to assess differences between sub-groups.

## 3. Results and Discussion

Of the 151 PCTs in England in financial year 2010/11, an annual return of service provision was available for 147. From this list 17 were selected, one reporting two commissioned services, for questionnaire development and piloting, and 16 for the questionnaire distribution. The latter reported 1 (4 PCTs), 2 (6), 3 (4) or ≥4 (2) commissioned services and included 778 pharmacies in total (Table 1).

### 3.1. Response Rate

The overall response rate was 206 (26.5%), ranging from 15.1% to 35.9% between PCTs (Table 1). Response rate was unrelated to the number of reported commissioned services. Two-thirds of responses were received after the first mailing (138; 67%), with a further 56 (27%) following the second mailing and 12 (6%) after the telephone reminder.

**Table 1:** Number of pharmacies, reported public health services commissioned and questionnaire return rate for the 16 Primary Care Trust areas in the study. NHS—National Health Services; PCT—Primary Care Trusts.

Number of NHS-reported public health services	Number of pharmacies in PCT	Number (%) of questionnaires returned
1	34	10 (29.4%)
	51	11 (21.6%)
Sub-total	85	21 (24.7%)
2	64	12 (18.8%)
	52	17 (32.7%)
	44	11 (25.0%)
	49	10 (20.4%)
Sub-total	209	50 (23.9%)
3	27	8 (29.6%)
	73	17 (23.4%)
	53	8 (15.1%)
	49	12 (24.5%)
	77	15 (19.5%)
	46	7 (15.2%)
Sub-total	325	67 (20.6%)
4	69	16 (23.2%)
	39	14 (35.9%)
	66	18 (27.3%)
	64	20 (31.3%)
Sub-total	238	68 (28.6%)
Total	778	206 (26.5%)

### 3.2. Demographic Characteristics

The characteristics of both the pharmacist responders and their pharmacies are shown in Table 2. Approximately half the respondents had qualified within the previous ten years, two-thirds were managers, 10% owners and 62% were working in multiples (defined as > 6 pharmacies). Twenty-nine (14.1%) indicated their pharmacy was designated as a Healthy Living Pharmacy (HLP), but 34 (16.5%) did not know and four (0.2%) did not respond. The 29 HLPs were across areas with differing levels of commissioned services and 23 were in multiple ownership.

**Table 2:** Characteristics of pharmacist responders and pharmacies (*n* = 206).

Characteristic		Number (Proportion of total)
Gender (203) *	Female	91 (44.8)
	Male	112 (55.2)
Years qualified (196) *	5 or fewer	49 (25.0)
	5 to 10	51 (26.0)
	More than 10	96 (49.0)
Role in pharmacy (205) *	Manager	134 (65.7)
	Second pharmacist	18 (8.8)
	Locum	17 (8.3)
	Superintendent	14 (6.9)
	Owner	22 (10.3)
Type of pharmacy	Multiple ( > 5 pharmacies)	128 (62.1)
	Independent pharmacy	78 (37.9)
Location of pharmacy	High street/suburban	80 (38.8)
	Shopping precinct/out of town centre	19 (9.3)
	Village	43 (20.9)
	Supermarket	14 (6.8)
	Attached to GP surgery	22 (10.7)
	Other (city centre, secondary parade, residential, other)	28 (13.6)

* Missing data excluded.

### 3.3. Public Health Service Provision

Respondents self-reported a median of six locally commissioned public health services (range 0 to 26; mean ± SD = 6.44 ± 3.8). The mean number of services respondents indicated they provided was not related to the number of commissioned services reported by the PCTs in which their pharmacy was located. Pharmacists from areas with only one PCT-reported public health service claimed they delivered a mean of 8.1 services; the equivalent for PCTs reporting two, three and four services was 7.2/pharmacy, 6.5/pharmacy and 5.3/pharmacy respectively. There was no statistically significant difference in the mean number of public health services provided by the 29 HLPs (6.7 ± 3.9) compared to the remaining pharmacies (6.3 ± 3.3) (*t* = 0.58; *p* = 0.56). Nor were there differences in the mean number of services provided by 78 pharmacies classed as independent (6.2 ± 3.6) compared to the 128 multiples (6.6 ± 3.9) (*t* = 0.65; *p* = 0.514).

**Figure 1:**
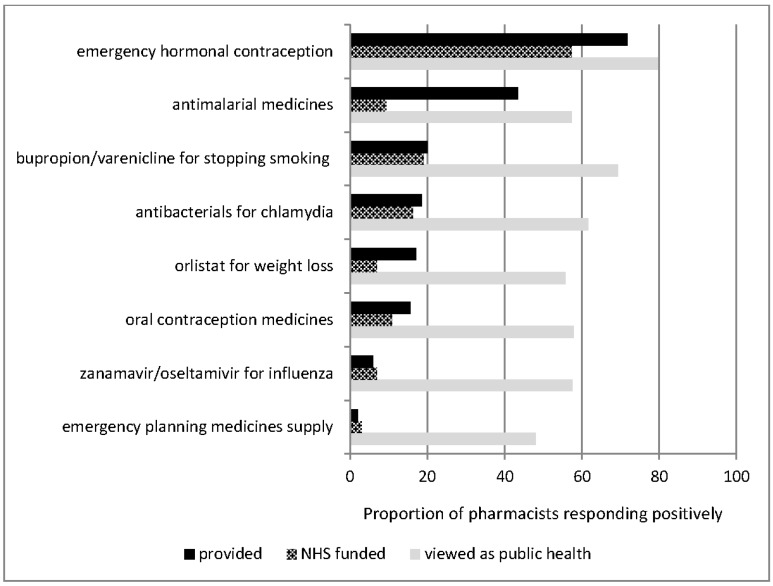
Self-reported provision of medicines supply under group direction.

The most common service provided through a patient group direction which was NHS-funded was emergency contraception, but some pharmacies provided other medicines which were non-NHS-funded, particularly antimalarial treatment and orlistat (Figure 1). However not all were viewed as public health, particularly medicines provision for emergency planning.

**Figure 2:**
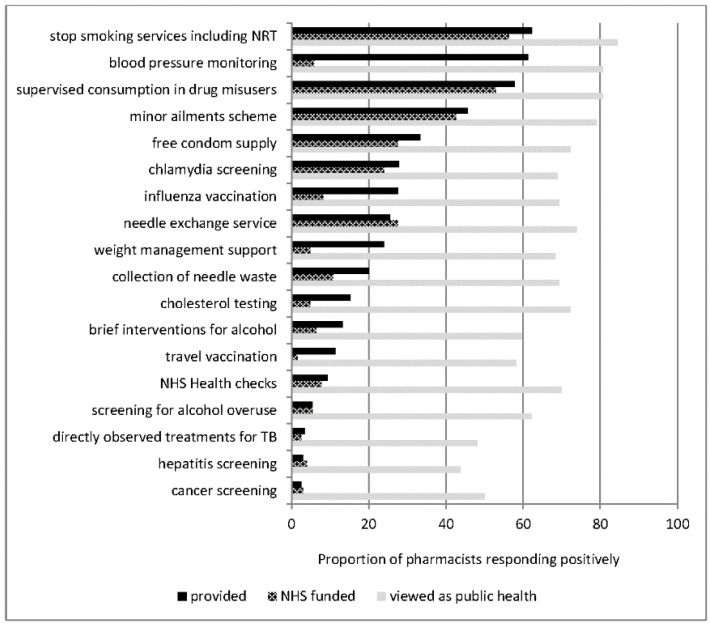
Self-reported provision of other public health services.

Among other services not involving medicines supply under a patient group direction, stop smoking support was most frequently provided, followed by blood pressure monitoring, although while the former was primarily NHS-funded, the latter was not (Figure 2). Other services provided which were reported as not being NHS-funded were: influenza vaccination, weight management support, cholesterol testing and travel vaccination.

The public health services perceived by pharmacist respondents as being most valued by customers were smoking cessation support (cited by 97/188; 51.6%), emergency contraception (72; 38.3%) and minor ailments (49; 26.1%), all NHS-funded services, with blood pressure monitoring also rated as being valued by 31 (16.5%). Forty-one respondents (19.9%) indicated a desire to provide more services, 30 of whom indicated examples of these, the most frequent being anticoagulant services (4; 13%).

### 3.4. Reasons for Providing Public Health Services

From the range of potential reasons for providing public health services included in the questionnaire, those most frequently cited were professional responsibility (151; 76.6%), patient satisfaction (147; 74.6%) and personal satisfaction (131; 66.5%). Personal satisfaction was cited more frequently by respondents from independently-owned pharmacies compared to multiples (55/73; 75.3% *vs*. 76/124; 61.3%; Pearson’s chi-square = 4.01; *p* = 0.03) and among owners/superintendents compared to managers/second pharmacists (28/33; 84.8% *vs*. 102/162; 63.0%; Pearson’s chi-square = 5.91; *p* = 0.01). Financial incentives were selected by relatively few, with profit margin chosen by only 46 (23.4%) and bonus payments by even fewer (28; 14.2%), with no differences relating to pharmacy ownership or pharmacist role. Pressure from head office was cited by 62, 57 of whom described their pharmacy as a multiple (Pearson’s chi-square = 32.6; *p* <0.001 compared to independent).

Requests from patients/the public was cited by approximately a third of respondents (66; 33.5%) as a reason for providing services, with a similar proportion (69; 35.2%) also selecting this as a reason for choosing which services to provide. More frequently cited reasons for choosing services were the needs of patients/the public (157; 80.1%) and competence (95; 48.5%). Profit margin and performance targets were again less important, selected by 50 (25.5%) and 57 (29.1%) respectively.

Respondents’ perceptions of who made decisions on which services to provide were most frequently that it was a combination of individuals/organisations (73; 35.4%), but many also considered these decisions were made entirely by the company/superintendent pharmacist (50; 24.3%) or by the PCT (26; 12.6%). Only 70 (34.0%) felt personally involved in decisions about service provision, most of whom (68) were either managers or owners/superintendents. Overall 82 (39.8%) considered the PCT were involved and only 11 (5.3%) the LPC.

There were 126 respondents (64.0%) who agreed/strongly agreed with the statement: “public health services are based on the needs of my local population”. However, awareness of documents outlining need for services ranged from 40.9% for local health profiles to 74.7% for PNAs (Table 3). Fewer than half claimed to have read these documents, with the PNA being most widely read, but fewer than 20% had used one of them to help decide on services, again the most common being the PNA. Overall slightly more owners/superintendents claimed to have read the PNA, but HLP status or number of PCT-commissioned services did not affect use of these documents.

**Table 3:** Awareness and use of local information relating to population needs (*n* = 206)

Document	Number (proportion) of pharmacists who…		
	Are aware of	Have read	Used to help decide on services
Pharmaceutical needs assessment (PNA)	148 (74.7%)	96 (48.5%)	33 (16.7%)
Joint Strategic Needs Assessment (JSNA)	104 (52.5%)	36 (18.2%)	11 (5.6%)
Direct of Public Health report	100 (50.5%)	29 (14.6%)	7 (3.5%)
Local health profile	81 (40.9%)	21 (10.6%)	7 (3.5%)
Pharmacy in England	119 (60.1%)	85 (42.9%)	10 (5.1%)
Choosing Health through Pharmacy	106 (53.5%)	51 (25.8%)	8 (4.0%)

### 3.5. Views on Commissioning, Promotion and Support for Public Health Services

Responses to the statements covering commissioning, promotion of and support for pharmacy-based public health services are shown in Table 4. The large majority of respondents were positive about the value of these services to both the public and to their pharmacies. In addition, responses to further statements showed that 148 respondents (75.1%) agreed/strongly agreed that: “public health services are vital for my pharmacy’s business” and 106 (53.5%) disagreed/strongly disagreed that: “I do not focus on public health services because dispensing volume is more important”. There were no differences in these proportions relating to pharmacy ownership, number of commissioned services or pharmacist role.

**Table 4:** Pharmacists’ views on current commissioning, promotion and support for pharmacy public health services.

Statement	Anchor	Frequency of responses (% of total responding)					Anchor
Pharmacy-based public health services are…	not valued by the public	2.6	10.7	21.4	37.8	27.6	valued by the public
The public is…	unaware of these services	8.6	24.7	40.9	20.2	5.6	aware of these services
Promotion of these services needs to …	increase	55.6	21.2	12.6	7.1	3.5	decrease
Promoting pharmacy-based public health services is …	a local responsibility	9.1	8.1	21.8	20.3	40.6	a national responsibility
Promotion of these services is the responsibility of…	the profession	8.6	11.7	43.7	18.8	17.3	the government
Promotion of these services is…	my responsibility	13.1	17.2	51.5	10.6	7.6	someone else’s responsibility
In my local area pharmacy public health services are…	increasing	13.1	26.3	36.9	11.1	12.6	decreasing
Providing public health services is…	a waste of my time	2.0	6.6	24.9	36.0	30.5	highly valuable for me
My local GPs are…	unsupportive of pharmacy based public health services	17.4	27.4	34.3	14.4	6.5	supportive of pharmacy public health services
The local director of public health is…	supportive of pharmacy based public health services	11.1	18.2	51.5	12.6	6.6	unsupportive of pharmacy public health services
The pharmacists at our PCT are the…	barrier to developing public health services	5.5	9.0	33.8	33.8	17.9	advocates of developing public health services
My Local Pharmaceutical Committee…	aids local service development	22.9	31.8	30.3	9.0	6.0	hinders local service development

A high proportion of respondents viewed local PCT staff as supportive, although fewer indicated that local GPs supported pharmacy public health services, with no differences dependent on the number of PCT-reported commissioned services. Most respondents (76.8%) also considered there was a need for increased promotion of services, but there was less clarity about whose role this was, with 60.9% indicating that responsibility was national, but 43.7% were unsure whether this responsibility lay with the profession or the government. Moreover, only 30.3% felt that promotion of pharmacy public health services was their responsibility, with just over half selecting the mid-point option. There was also uncertainty about the extent to which the Director of Public Health was supportive.

### 3.6. Future Development of Public Health Services

Most respondents (69.9%) felt they would need a lot of support during the period of change to service commissioning, with only 14.8% indicating they felt fully informed about the changes to the NHS. However, 69.8% indicated that the NHS changes could put the commissioning of pharmacy public health services at risk (Table 4). Results suggest uncertainty about whether these changes would increase or decrease opportunities to provide public health services and whether local or national support would be needed.

Public health services were viewed as being of increasing importance in the future, with 44.8% of respondents indicating they were looking forward to public health services being the main focus of their work, 83.7% being of the view that provision of public health advice would increase in future and 54.2% that profit from public health services would be more important than profit from dispensing in future (Table 5). Moreover, 150 (72.1%) also agreed/strongly agreed with the statement: “I would focus on public health services more if demand from the public increased”.

### 3.7. Summary of Main Findings

This survey involved community pharmacists in 16 areas spread throughout England, selected on the basis of differing numbers of PCT-reported locally commissioned public health services. It is the first national survey to seek pharmacist views on reasons for providing public health services and the local and national support for these. Respondents self-reported offering a wider range of services than indicated by NHS data, indeed the number of self-reported services was unrelated to the number in NHS returns. There were no differences in the number of services provided dependent on pharmacy ownership or HLP status, although the latter were few in number. Smoking cessation, blood pressure monitoring and supervised drug consumption were provided by over 50% of respondents. Blood pressure monitoring was identified as one a service which was highly valued by customers, but was not generally offered as an NHS-funded service.

**Table 5:** Pharmacists’ views on NHS reorganisation and the future of pharmacy public health services.

Statement	Anchor	Frequency of responses (% of total responding)					Anchor
The NHS is about to undergo major reorganisation and I feel…	uninformed about the proposed changes	15.3	34.5	35.5	12.8	2.0	fully informed about these changes
With the coming changes in local commissioning of public health services in my area I will require...	little support to help me through these changes	2.5	5.4	22.2	37.9	32.0	a lot of support to help me through these changes
During this time of change in the NHS I will look for support from…	local pharmacy bodies such as the LPC	18.3	15.8	35.1	17.3	13.4	national pharmacy bodies such as the RPS or PSNC
I believe the commissioning of pharmacy based public health services...	will be unaffected by the planned changes to the NHS	1.0	4.0	25.2	33.7	36.1	is at risk as a result of planned changes in the NHS
Changes in commissioning of pharmacy services may...	remove opportunities for me to develop my public health practice	13.4	18.3	40.6	19.8	7.9	provide further opportunities for me to develop my public health practice
If demand for these services increased…	I would not cope with the extra work	9.1	21.7	28.8	21.7	18.7	I would welcome the extra work
I am looking forward to public health services being …	removed from my day to day work to let me focus on dispensing	1.0	7.5	46.8	26.9	17.9	the main focus of my day to day work now that dispensing can be undertaken by qualified technicians
My role in dispensing medicines for patients will	increase	10.3	12.3	26.6	34.5	16.3	decrease
My role in providing health advice to the public	increase	38.9	44.8	8.9	5.4	2.0	decrease
Profit from these services will be…	less important that profit from dispensing	3.4	8.9	33.5	27.6	26.6	more important than profit from dispensing
My role in provision of these services	increase	39.1	40.1	14.9	4.0	2.0	decrease

Motivation for providing public health services was professional not financial, particularly in independent pharmacies. However only 35% indicated personal involvement in decisions regarding which services should be delivered, with less than a fifth of pharmacists having used local public health reports to inform these decisions. While most respondents were positive about providing public health services and viewed them as potentially increasing in future, there were mixed views concerning local and national support for service provision and promotion, plus a desire for support in relation to changes which could affect service commissioning. Many respondents selected neutral options in relation to statements concerning promotion of, support for and the future of pharmacy public health services. This may be a reflection of the lack of awareness of local priorities, the content of local documents and what impact the re-structuring would have on service commissioning.

### 3.8. Strengths and Limitations

The questionnaire used was developed using data from interviews conducted with community pharmacists providing public health services, thus covered issues relevant to service commissioning and provision. We did not seek views on the provision of privately-funded public health services. The survey included all pharmacies in a stratified random sample of PCTs, which ensured that for the areas included the number of commissioned services varied. However, only 16 of the 151 total PCTs in England at the time of the study, thus findings may not be nationally representative. All pharmacies within each PCT were included in the survey, but it achieved a low response rate, despite two reminders. Respondents were thus potentially biased towards providers of public health services, perhaps also with positive views. Service provision and use of documents were self-reported, thus subject to potential social desirability and recall bias. Our definition of multiple pharmacy ownership was six or more pharmacies, thus our findings are not directly comparable to other work using different ownership options.

### 3.9. Relation to Literature/Implications for Policy and Practice

The findings confirm those of many studies reporting pharmacists’ views towards individual public health services, in that the profession in England has positive views about public health [18,19]. Our work differs from most previous work in that the target population was all pharmacists in areas where any public health services were commissioned, rather than those known to be providing particular services. One survey involving a random sample of Scottish community pharmacists conducted in 2007 found 79% of the 223 responders agreed that public health was important to their practice [21]. Frequency of service provision was similar to previous surveys, [12,13] with smoking cessation support and supervised consumption for drug misusers being the most common public health services and emergency contraception being the most frequent supply under PGD. The lack of a relationship between self-reported service provision and PCT-reported service commissioning may be due to the lack of specificity within the NHS General Pharmaceutical reporting framework, which requires only provision of services under PGD and screening, lacking detail of which services are commissioned. This does not allow a comprehensive picture of service provision nationally. Moreover PCTs were not required to provide information about some fairly widespread services, such as weight management and vaccinations.

Interestingly, many pharmacist respondents did not view some of these services as public health, particularly some involving PGDs and screening, which were provided by relatively few pharmacists. This is somewhat surprising and may indicate that views of a service are influenced by its provision.

Over a third of the 1023 respondents to a previous large comprehensive national (Great Britain) survey felt conflicts with commercial interests were a barrier to providing public health services, and the findings indicated showed that service provision varied dependent on pharmacy ownership [13]. This study concluded that large companies held an advantageous position with regard to attracting funding for these services, with the potential that independent pharmacies may become less able to provide them. Other work, which was a pragmatic evaluation of the HLP programme, suggested that HLPs were offering more services than other pharmacies and that service uptake increased in pharmacies after becoming HLPs [16]. In contrast, our study showed no significant differences in the number of services reported as being provided, depending on either pharmacy ownership or HLP designation, however the number of HLPs was small and the categorisation of ownership limited.

Our results suggest that decisions on which services to provide frequently do not involve the pharmacists delivering them, with health organisations, such as PCTs and commercial companies/managers being most likely to decide on services. Individual pharmacists had little awareness of local documents which would inform these decisions and, while PCTs and successor organisations commissioning services are likely to base decisions on the loc al needs described in these documents, the extent to which pharmacy companies do so is not known. Our survey did not determine whether pharmacists felt constrained by their lack of involvement in decision-making, but there were a significant number who indicated a desire to provide more services than at present.

The majority of respondents expected public health services to increase in future at the expense of dispensing. However many also showed concerns for their future development, given the changes which have since taken place within primary care. This could be related to the perceptions that pharmacists working in PCTs and LPCs were key to service development, with GPs and public health directors being viewed as less supportive. Nonetheless these results must be viewed with caution, due to the low response rate. The changes implemented in April 2013 mean that GPs now have greater influence over service commissioning than at the time of this survey. There is anecdotal evidence of service commissioning bypassing LPCs and services being de-commissioned, while more recent annual NHS returns show the overall number of services provided has reduced.

## 4. Conclusions

The wide range of services provided and the positive expectations of pharmacists towards increased provision of public health services are important for the future development of these services. Greater involvement in decision making and awareness of local needs may enable pharmacists to tailor services more effectively. Support from both national and local organisations is needed for future commissioning and provision of pharmacy public health services.

## References

[1] Department of Health (1999). Saving Lives: Our Healthier Nation.

[2] Department of Health (2003). Tackling Health Inequalities: A Programme for Action.

[3] Department of Health (2005). Choosing Health through Pharmacy: A Programme for Pharmaceutical Public Health 2005–2015.

[4] Department of Health (2008). Pharmacy in England: Building on Strengths—Delivering the Future.

[5] Department of Health (2010). Healthy Lives, Healthy People: Our Strategy for Public Health in England.

[6] Clucas K. (1986). Pharmacy: The Report of a Committee of Inquiry Appointed by the Nuffield Foundation.

[7] Royal Pharmaceutical Society of Great Britain (1995). Pharmacy in a New Age: Developing Strategy for the Future of Pharmacy.

[8] Department of Health (2004). Choosing Health: Making Healthy Choices Easier.

[9] Pharmaceutical Services Negotiating Committee (2013). The Vision for NHS Community Pharmacies: The Path to Improved Patient Care.

[10] Smith J., Picton C., Dayan M. (2013). Now or Never: Shaping Pharmacy for the Future.

[11] Blenkinsopp A., Bond C.M., Celino G., Inch J., Gray N. (2007). National Evaluation of the New Community Pharmacy Contract.

[12] Bush J., Langley C.A., Jesson J.K., Wilson K.A. (2006). Perceived barriers to the development of community pharmacy’s public health function: A survey of the attitudes of directors of public health and chief pharmacists in UK Primary care organisations. Int. J. Pharm. Pract..

[13] Bush J., Langley C.A., Wilson K.A. (2009). The corporatization of community pharmacy: Implications for service provision, the public health function, and pharmacy’s claims to professional status in the United Kingdom. Res. Soc. Admin. Pharm..

[14] Pharmaceutical Services Negotiating Committee Healthy Living Pharmacies. http://psnc.org.uk/services-commissioning/locally-commissioned-services/healthy-living-pharmacies/.

[15] Brown D., Portlock J., Rutter P., Nazar Z. (2014). From community pharmacy to health living pharmacy: Positive early experiences from Portsmouth, England. Res. Soc. Admin. Pharm..

[16] Duggan C., Evans D., Holden M., Kennington E., Leach R., Root G., Shepherd E. (2013). Evaluation of the Healthy Living Pharmacy Pathfinder Work Programme 2011–2012.

[17] NHS England (2014). Understanding the new NHS. http://www.england.nhs.uk/wp-content/uploads/2014/06/simple-nhs-guide.pdf.

[18] Eades C.E., Ferguson J.S., O’Carroll E. (2011). Public Health in community pharmacy: A systematic review of pharmacist and consumer views service. BMC Publ. Health.

[19] Anderson C., Blenkinsopp A., Armstrong M. (2003). Pharmacists’ perceptions regarding their contribution to improving the public’s health: A systematic review of the United Kingdom and international literature 1990–2001. Int. J. Pharm. Pract..

[20] Health and Social Care Information Centre. http://www.hscic.gov.uk.

[21] Pfleger D.E., McHattie L.W., Diack H.L., McCaig D.L., Stewart D.C. (2008). Views, attitudes and self-assessed training needs of Scottish community pharmacists to public health practice and competence. Pharm. World Sci..

